# Field evaluation of tissue culture-derived and offshoot-grown date palm cultivars: a comparative analysis of vegetative and fruit attributes

**DOI:** 10.3389/fpls.2025.1516983

**Published:** 2025-04-24

**Authors:** Najamuddin Solangi, Abdul Aziz Mirani, Mushtaque Ahmed Jatoi, Adel Ahmed Abul-Soad, Lal Bux Bhanbhro, Ghulam Sarwar Markhand, Mohammad Hedayat, Gholamreza Abdi

**Affiliations:** ^1^ Date Palm Research Institute, Shah Abdul Latif University, Khairpur, Pakistan; ^2^ Horticulture Research Institute, Agricultural Research Centre, Giza, Egypt; ^3^ Department of Chemistry, Shaheed Benazir Bhutto University, Nawabshah, Pakistan; ^4^ Department of Biotechnology, Persian Gulf Research Institute, Persian Gulf University, Bushehr, Iran; ^5^ Department of Horticulture, College of Agriculture, Persian Gulf University, Bushehr, Iran

**Keywords:** field evaluation, tissue culture-derived, offshoot-grown, date palm cultivars, juvenile inflorescence, principal component analysis, K-means clustering, genetic stability

## Abstract

An analysis of the field performance of tissue culture (TC)-derived commercial cultivars of date palm (i.e., Kashuwari, Gulistan, and Dedhi) obtained from juvenile inflorescence explants was carried out to compare the different vegetative and fruit attributes with their respective offshoot (OS)-grown mother plants. A comparative analysis of leaf-, bunch-, and fruit-related variables was conducted 5 years after planting in an open field. The data obtained were used to perform ANOVA, *k*-means clustering, and principal component analysis (PCA). The results revealed that the majority of the variables showed non-significant variations between TC plants and OS-grown mother plants across all three cultivars. *K*-means clustering produced three distinct clusters for each of the three cultivars, placing all their TC and OS plants together in their respective clusters, except for one TC plant of cv. Gulistan, which was placed with cv. Dedhi. The PCA results showed that the first two components explained a significant proportion of the total variation in all three date palm cultivars, ranging from 71.4% to 76.4%. This study provides critical insights for the validation of TC methods, assessing adaptability under open field conditions, ensuring genetic stability, and ultimately expanding the adoption and impact of TC techniques in agriculture.

## 1 Introduction

The date palm (*Phoenix dactylifera* L.) is a monocotyledonous, perennial, diploid (2*n* = 36), dioecious tree that belongs to the family Arecaceae (Abul-Soad, 2011; Solangi et al., 2023a, 2024). It has been estimated that 150 million date palm trees and 5,000 varieties exist around the world, which are classified based on their fruit characteristics, such as fruit size, shape, color, sugar and moisture content (Solangi et al., 2022). Date palm is one of the most economically valuable fruit crops (Harkat et al., 2022). It contributes to environmental conservation, agricultural sustainability, and food security in arid regions of the world with limited water resources (Haj-Amor et al., 2020; Dhaouadi et al., 2021). The global cultivated area of date palms in 2021 was 1.30 million hectares, producing 9.66 million tons (FAOSTAT, 2023). The date fruit is a rich source of vitamins, minerals, and sugars and represents the most economically important product of date palm cultivation (Ahmed et al., 2013; Al-Mayahi, 2024). Pakistan is ranked among the major date-producing and exporting countries, with cultivation thought to date back to 5,000 BC (Jatoi et al., 2015). The date palm cultivars Dedhi, Gulistan, and Kashuwari are among the commercially important soft and semi-dry cultivars from Pakistan. However, the population of soft and semi-dry date cultivars in the region is declining due to various factors such as diseases and pests (Maitlo et al., 2013), the conversion of date palm orchards to housing societies (Abul-Soad et al., 2017), and preference of farmers to grow exotic cultivars (Abul-Soad et al., 2013). In addition, the lack of proper processing and storage facilities and the inadequate marketing channels for both local and international exports of soft or semi-dry dates in Pakistan have contributed to the shift in cultivation from soft and semi-dry to dry date palm cultivars (Abul-Soad et al., 2017). Consequently, the availability of offshoots (OS) of the soft and semi-dry date palm cultivars for commercial plantations is sometimes limited. Specifically, the propagation of cv. Dedhi and cv. Kashuwari using OS is challenging due to the limited number of available OS and the low survival rate of OS in the field (Mirani, 2019).

Tissue culture (TC) represents a sustainable alternative to traditional propagation, which addresses the demand–supply gap by producing genetically stable plants with enhanced adaptability to field conditions. This technique also plays a critical role in the preservation of elite cultivars facing extinction due to urbanization and disease. In date palms, TC is an alternative to the traditional OS transplantation method for the production of genetically stable and homogeneous palms, which helps meet the demand of approximately 1–2 million plants per year in the international date palm market. However, somaclonal variation (SV), or changes in the vegetative, reproductive, and/or fruit quality in TC date palms, remains a challenging issue. Detection of SVs using phenotypic (Mirani et al., 2019) and molecular markers (Mirani et al., 2020) or field evaluation of important vegetative- or fruit-related variables is necessary to confirm the true-to-typeness and fruiting quality of TC plants. Furthermore, phenotypic attributes are also useful for the assessment of agronomic performance (Bhanbhro et al., 2019) or the detection of quantitative trait loci (QTLs) (Bux et al., 2019) in various crops.

In the current study, TC plants of three date palm cultivars (i.e., Kashuwari, Gulistan, and Dedhi) derived from juvenile inflorescence explants at the Date Palm Research Institute (DPRI), Shah Abdul Latif University (SALU), Pakistan, were planted in a nursery for field evaluation (Jatoi et al., 2015). The genotypic nature of the produced plants was already studied by Mirani et al. (2019); Mirani et al. (2020), and Mirani et al. (2022) using phenotypic and transposable element-based molecular markers. This study hypothesized that TC-derived plants would exhibit phenotypic traits similar to those of OS-grown plants due to their genetic identity, although minor variations may arise due to juvenility or SVs. This study aimed to assess the extent of these similarities and differences in the vegetative, bunch, and fruit characteristics.

## 2 Materials and methods

### 2.1 Vegetative characteristics

Leaf length (LL), length of spined part (LSP), length of pinnated part (LPP), pinnated part ratio (PPR), number of spines (NS), number of pinnae (NP), length of pinnae(LP), and width of pinnae (WP) were determined according to Mirani (2019).

PPR was calculated using following formula:


$$PPR=\;\frac{LPP\times 100\;}{LL}$$

### 2.2 Bunch characteristics

Bunch length (BL), number of strings per bunch (NSB), total number of fruits per bunch (TNFB), and percentage of retained fruits (RF%) were calculated according to Mirani (2019).

RF% was calculated using following formula:


RFB=NSB×RFS

RFB and RFS are retained fruits per bunch and retained fruits per string, respectively.

### 2.3 Fruit physical characteristics

Fruit length (FL), fruit diameter (FD), fruit weight (FW), pulp weight (PW), fruit-to-pulp ratio (FPR), seed length (SL), seed diameter (SD), and seed weight (SW) were determined according to Mirani (2019). FPR was calculated using the following formula:


$$PFR=\;\frac{PW\;}{FW}\times 100$$

SW was calculated using the following formula:


SW = FW – PW

FW and PW are the fruit weight and pulp weight, respectively.

### 2.4 Phenotypic data analysis

To standardize the measurements of vegetative and bunch characteristics across propagation methods and cultivars, data were collected from both TC-derived plants (5 years old) and OS-grown plants (20–40 years old) to ensure that all plants were at comparable phenological stages. Measurements were taken during the same growing season using calibrated instruments, such as digital calipers and tape measures, by the same trained personnel in order to minimize observer bias. The plants were grown under uniform agronomic practices, such as consistent irrigation and fertilization, to ensure uniformity. For each cultivar, data were recorded from seven TC plants and one OS plant, with each trait measured in triplicate and then averaged to reduce variability. Traits that are prone to high variability, such as fruit retention or bunch weight, were normalized using percentages or ratios relative to plant size or weight. These methods ensured consistency and reliability in the comparison of traits across cultivars and plant types.

The obtained phenotypic data for the vegetative, bunch, and fruit physical characteristics were processed for ANOVA using SPSS software (version 20.0). Differences between means were calculated using the least significant difference (LSD) at the ≤0.05 level of significance (Steel and Dickey, 1997).

### 2.5 *K*-means clustering and principal component analysis

*K*-means clustering was performed for plant groups based on phenotypic similarities, providing insight into trait variability within and across cultivars. Principal component analysis (PCA) was used to reduce data dimensionality, prioritizing components that explained the highest variance (>70%), enabling better visualization and identification of key traits contributing to the differences.

*K*-means clustering (Hartigan and Wong, 1979) was performed using the “k-means” function from the “factoextra” R package (Kassambara, 2016). The dataset, which consisted of 21 phenotypic (vegetative, bunch, and fruit) variables of the three date palm cultivars, was preprocessed by scaling using the “scale” function. The Euclidean distance metric was employed to calculate the pairwise distances between data points. The optimal number of clusters (*k*) was determined using the elbow method (Sugar and James, 2003) and was visualized with the “fviz_nbclust” function. The *k*-means algorithm was applied with four initializations (“nstart = 100”) to enhance robustness. Clustering results were obtained through the assignment of data points to the clusters based on minimizing the within-cluster sum of squares. The final clustering results were visualized using the “fviz cluster” function.

The phenotypic data (vegetative, bunch, and fruit) of all three date palm cultivars were used separately in this study. The structure of the dataset was examined using the “str()” function in the R package. Numerical data, which consisted of 21 variables, were extracted for further analysis. The data were normalized using the “scale()” function to standardize the variables. A correlation matrix was computed for the normalized data and was visualized using the “ggcorrplot” package. PCA was performed on the correlation matrix to capture the underlying patterns in the data (Xie, 2019). Eigenvalues were visualized to identify the proportion of variance explained by each principal component (PC). The loadings and contributions of the variables to the PCs were examined and visualized to understand the relationships between the variables.

## 3 Results

### 3.1 Vegetative characteristics of TC and OS plants

**Table 1** shows the mean variations in the vegetative characteristics of the TC and OS (mother) plants of date palm cultivars Kashuwari, Gulistan, and Dedhi. The data indicated that, in terms of LL, no significant difference (LSD < 0.05) was observed between the TC and OS plants of date palm cv. Kashuwari. TC plants exhibited an LL of 121.71 in., while OS plants had an LL of 130.29 in. Similarly, no significant differences were found between the TC and OS plants in terms of LSP, LPP, PPR, and NS. However, OS plants showed significantly higher NP (211.86) compared with TC plants (194.57). Similarly, the LP and WP were also significantly higher in OS plants than in TC plants. The date palm cv. Gulistan showed a significant difference in only one vegetative characteristic between the TC and OS plants. The data in **Table 1** show that the OS plants exhibited a significantly higher LL (155 in.) than the TC plants (140.43 in.). In all other noted vegetative characteristics, such as LSP, LLP, PPR, NS, NP, LP, and WP, the data showed no significant difference (LSD < 0.05) between the TC and OS plants.

**Table 1:** Comparison of the vegetative characteristics of tissue culture (TC)-derived plants and offshoot (OS)/mother plants of date palm cultivars Kashuwari, Gulistan, and Dedhi.

Cultivar	Plant type	LL (in.)	LSP (in.)	LPP (in.)	PPR (%)	NS	NP	LP (cm)	WP (cm)
Kashuwari	OS	130.29 ± 3.9a	34.14 ± 0.97a	96.14 ± 3.75a	73.65 ± 0.98a	19.86 ± 0.99a	211.86 ± 4.38a	42.82 ± 0.55a	3.57 ± 0.03a
	TC	121.71 ± 3.9a	34.57 ± 0.97a	87.14 ± 3.75a	71.51 ± 0.98a	18 ± 0.99a	194.57 ± 4.38b	40.96 ± 0.55b	3.29 ± 0.03b
Gulistan	OS	155 ± 4.29a	28.29 ± 1.28a	126.71 ± 3.74a	81.79 ± 0.75a	12.29 ± 0.76a	163.14 ± 3.84a	48.86 ± 0.92a	2.74 ± 0.08a
	TC	140.43 ± 4.29b	25 ± 1.28a	115.43 ± 3.74a	82.12 ± 0.75a	10.71 ± 0.76a	167.57 ± 3.84a	46.43 ± 0.92a	2.69 ± 0.08a
Dedhi	OS	140 ± 1.95a	33.29 ± 0.82a	106.71 ± 1.88a	76.21 ± 0.61a	16 ± 1.1a	147.14 ± 2.16a	51.53 ± 1.27a	3.76 ± 0.12a
	TC	133.43 ± 1.95b	32.86 ± 0.82a	100.57 ± 1.88b	75.36 ± 0.61a	15.43 ± 1.1a	143.86 ± 2.16a	44.99 ± 1.27b	3.43 ± 0.12a
LSD	Kashuwari	0.15 n.s	0.76 n.s	0.12 n.s	0.15 n.s	0.21 n.s	0.02*	0.03*	<0.0001***
	Gulistan	0.03*	0.09 n.s	0.05*	0.76 n.s	0.17 n.s	0.43 n.s	0.09 n.s	0.6 n.s
	Dedhi	0.03*	0.72 n.s	0.04*	0.34 n.s	0.72 n.s	0.3 n.s	0.001**	0.07 n.s

Means followed by different lowercase letters within columns for each cultivar are significantly different (LSD test: *p* ≤ 0.05).

*LL*, leaf length; *LSP*, length of spined part; *LPP*, length of pinnated part; *PPR*, pinnated part ratio; *NS*, number of spines; *NP*, number of pinnae; *LP*, length of pinnae; *WP*, width of pinnae; *n.s.*, not significant.

*p ≤ 0.05; **p ≤ 0.01; ***p ≤ 0.001.

In the date palm cv. Dedhi, significant differences were found in LL, LPP, and LP, where OS plants showed significantly higher LL (140 in.), LPP (106.71 in.), and LP (51.53 cm) compared with 133.43 in., 100.57 in., and 44.99 cm in TC plants, respectively. On the other hand, there were no significant differences in the LSP, PPR, NS, NP, and WP between the TC and OS plants of date palm cv. Dedhi.

### 3.2 Bunch characteristics of TC and OS plants

**Table 2** shows the mean variations in the bunch characteristics of the TC and OS plants of date palm cultivars Kashuwari, Gulistan, and Dedhi. The data indicated that, in terms of BL, NSB, TNFB, and RF%, no significant difference (LSD < 0.05) was observed between the TC and OS plants of date palm cv. Kashuwari. Similarly, in date palm cv. Gulistan, all of the four noted bunch characteristics revealed no significant differences between the TC and OS plants at LSD < 0.05.

**Table 2:** Comparison of the bunch characteristics of tissue culture (TC)-derived plants and offshoot (OS)/mother plants of date palm cultivars Kashuwari, Gulistan, and Dedhi.

Cultivar	Plant type	BL (in.)	NSB	TNFB	RF%
Kashuwari	OS	48.1 ± 2.56a	65.29 ± 1.97a	1,608.06 ± 107.91a	65.52 ± 3.06a
	TC	43.62 ± 2.56a	62.71 ± 1.97a	1,575.37 ± 107.91a	61.25 ± 3.06a
Gulistan	OS	82.24 ± 5.08a	70.29 ± 4.22a	2,267.03 ± 143.72a	55.59 ± 1.92a
	TC	74 ± 5.08a	67.14 ± 4.22a	1,877.83 ± 143.72a	60.84 ± 1.92a
Dedhi	OS	96.58 ± 3.19a	73.71 ± 5.01a	2,128.4 ± 145.94a	62.52 ± 1.31a
	TC	86.57 ± 3.19b	70.86 ± 5.01a	2,079.2 ± 145.94a	57.18 ± 1.31b
LSD	Kashuwari	0.24 n.s	0.37 n.s	0.83 n.s	0.34 n.s
	Gulistan	0.27 n.s	0.61 n.s	0.08 n.s	0.08 n.s
	Dedhi	0.05*	0.69 n.s	0.82 n.s	0.01**

Means followed by different lowercase letters within columns for each cultivar are significantly different (LSD test: *p* ≤ 0.05).

*BL*, bunch length; *NSB*, number of strings per bunch; *TNFB*, total number of fruits per bunch; *RF%*, retained fruit percentage; *n.s.*, not significant.

*p ≤ 0.05; **p ≤ 0.01.

In contrast, in date palm cv. Dedhi, significant differences were recorded between the TC and OS plants in terms of BL and RF%. The data in **Table 2** show that the OS plants exhibited significantly higher BL (96.58 in.) and RF% (62.52%) compared with the 86.57-in. BL and 57.18% RF% in the TC plants. However, there were no significant differences in the NSB and TNFB between the TC and OS plants of cv. Dedhi.

### 3.3 Fruit characteristics of TC and OS plants

**Table 3** shows the mean variations in the fruit physical characteristics of the TC and OS plants of date palm cultivars Kashuwari, Gulistan, and Dedhi. Examination of the data obtained for the fruit physical attributes of Kashuwari palms detected no significant differences between OS and TC plants in terms of FL, FD, FW, PW, and FPR (F/PR%). Both propagation methods produced comparable fruit dimensions and composition. Similarly, SL, SD, and SW did not exhibit significant variations between OS and TC plants. The LSD test results confirmed the absence of significant differences in all measured parameters.

**Table 3:** Comparison of the fruit physical characteristics of tissue culture (TC)-derived plants and offshoot (OS)/mother plants of date palm cultivars Kashuwari, Gulistan, and Dedhi.

Cultivar	Plant type	FL (cm)	FD (cm)	FW (g)	PW (g)	FPR (%)	SL (cm)	SD (cm)	SW (g)
Kashuwari	OS	4.12 ± 0.18a	2.04 ± 0.04a	11.47 ± 0.47a	10.19 ± 0.46a	88.73 ± 0.58a	2.61 ± 0.06a	0.8 ± 0a	1.28 ± 0.05a
	TC	3.97 ± 0.18a	1.97 ± 0.04a	10.88 ± 0.47a	9.6 ± 0.46a	88.13 ± 0.58a	2.57 ± 0.06a	0.79 ± 0a	1.28 ± 0.05a
Gulistan	OS	4.53 ± 0.11a	2.16 ± 0.05a	12.84 ± 0.87a	11.43 ± 0.83a	88.71 ± 0.75a	2.6 ± 0.04a	0.91 ± 0.02a	1.41 ± 0.09a
	TC	4.11 ± 0.11b	1.97 ± 0.05b	11.14 ± 0.87a	9.8 ± 0.83a	87.9 ± 0.75a	2.46 ± 0.04b	0.89 ± 0.02a	1.34 ± 0.09a
Dedhi	OS	4.39 ± 0.08a	2.43 ± 0.02a	14.2 ± 0.53a	12.61 ± 0.51a	88.73 ± 0.6a	3 ± 0.04a	0.95 ± 0.03a	1.59 ± 0.07a
	TC	4.24 ± 0.08a	2.38 ± 0.02a	13.8 ± 0.53a	12.21 ± 0.51a	88.44 ± 0.6a	2.9 ± 0.04a	0.94 ± 0.03a	1.58 ± 0.07a
LSD	Kashuwari	0.55 n.s	0.26 n.s	0.39 n.s	0.38 n.s	0.48 n.s	0.64 n.s	0.18 n.s	0.39 n.s
	Gulistan	0.02*	0.01**	0.19 n.s	0.19 n.s	0.46 n.s	0.04*	0.36 n.s	0.56 n.s
	Dedhi	0.21 n.s	0.15 n.s	0.6 n.s	0.59 n.s	0.74 n.s	0.13 n.s	0.94 n.s	0.94 n.s

Means followed by different lowercase letters within columns for each cultivar are significantly different (LSD test: *p* ≤ 0.05).

*FL*, fruit length; *FD*, fruit diameter; *FW*, fruit weight; *FPR*, fruit-to-pulp ratio; *SL*, seed length; *SD*, seed diameter; *SW*, seed weight; *n.s.*, not significant.

*p ≤ 0.05; **p ≤ 0.01.

For date palm cv. Gulistan, significant differences (LSD < 0.05) were observed in certain fruit parameters between OS and TC plants. The OS plants showed longer fruits (FL), larger FD, and longer seeds (SL) compared with the TC plants. The OS plants had FL of 4.52 cm, FD of 2.16 cm, and SL of 2.6 cm compared with 4.11, 1.97, and 2.46 cm for the same parameters, respectively, in the TC plants. The FW, PW, and FPR, along with the seed parameters (SL, SD, and SW), showed no significant differences between the two propagation methods.

In the case of date palm cv. Dedhi, no significant differences were observed in the data (**Table 3**) between OS and TC plants for both fruit parameters (FL, FD, FW, PW, FPR) and seed parameters (SL, SD, SW). The two propagation methods produced similar results in terms of fruit and seed characteristics. The LSD test (<0.05) was used to identify significant differences among the traits due to its robustness in detecting subtle variations within small sample sizes. The non-significant results across the majority of traits reinforced the genetic stability of the TC plants, validating the TC protocol.

### 3.4 K-means clustering of OS and TC plants

**Figure 1:**
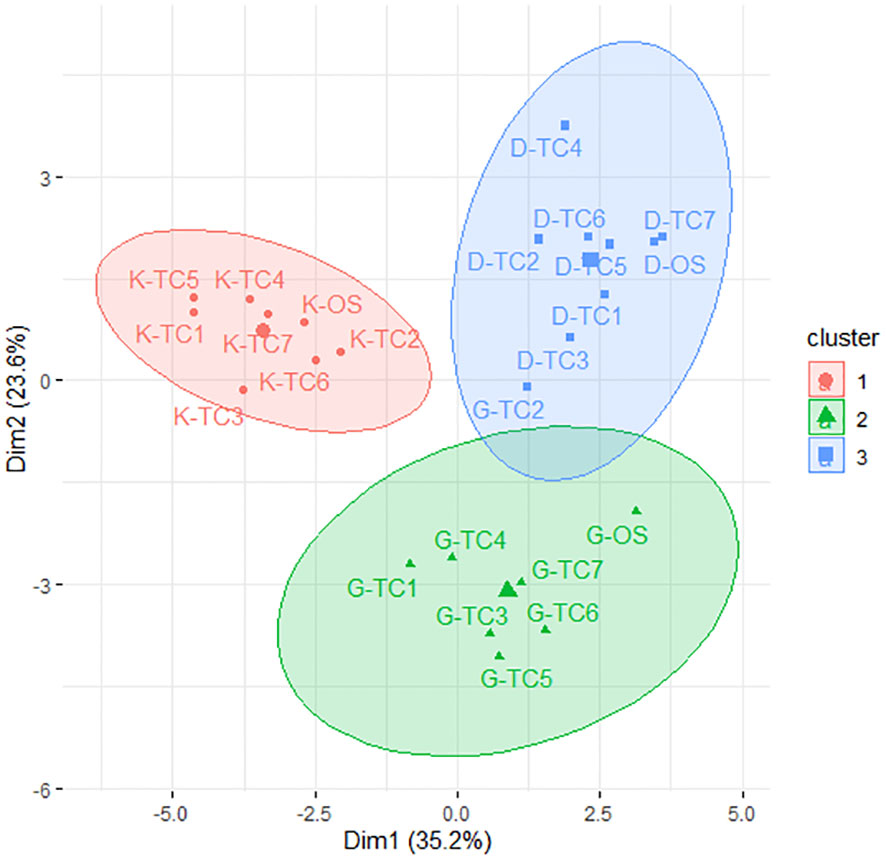
*K*-means clustering of the offshoot (OS)-grown and tissue culture (TC)-derived date palm plants of cultivars Dedhi, Kashuwari, and Gulistan. *D-TC*, Dedhi TC-derived plant; *D-OS*, Dedhi OS-grown plant; *K-TC*, Kashuwari TC-derived plant; *K-OS*, Kashuwari OS-grown plant; *G-TC*, Gulistan TC-derived plant; *G-OS*, Gulistan OS-grown plant.

*K*-means clustering analysis was performed on the vegetative, bunch, and fruit data of the TC and OS plants belonging to three date palm cultivars (i.e., Dedhi, Gulistan, and Kashuwari). For each cultivar, seven TC plants, along with one OS plant, were selected for the clustering analysis. **Figure 1** shows that all of the TC and OS plants from the three date palm cultivars can be divided into three distinct clusters: cluster 1 contained all seven TC plants from Kashuwari along with the OS plant (control), cluster 2 contained six TC plants from Gulistan along with the control, and cluster 3 contained all seven TC plants from Dedhi along with its control plant, and one TC plant from Gulistan.

### 3.5 PCA of the vegetative, bunch, and fruit data

**Figure 2:**
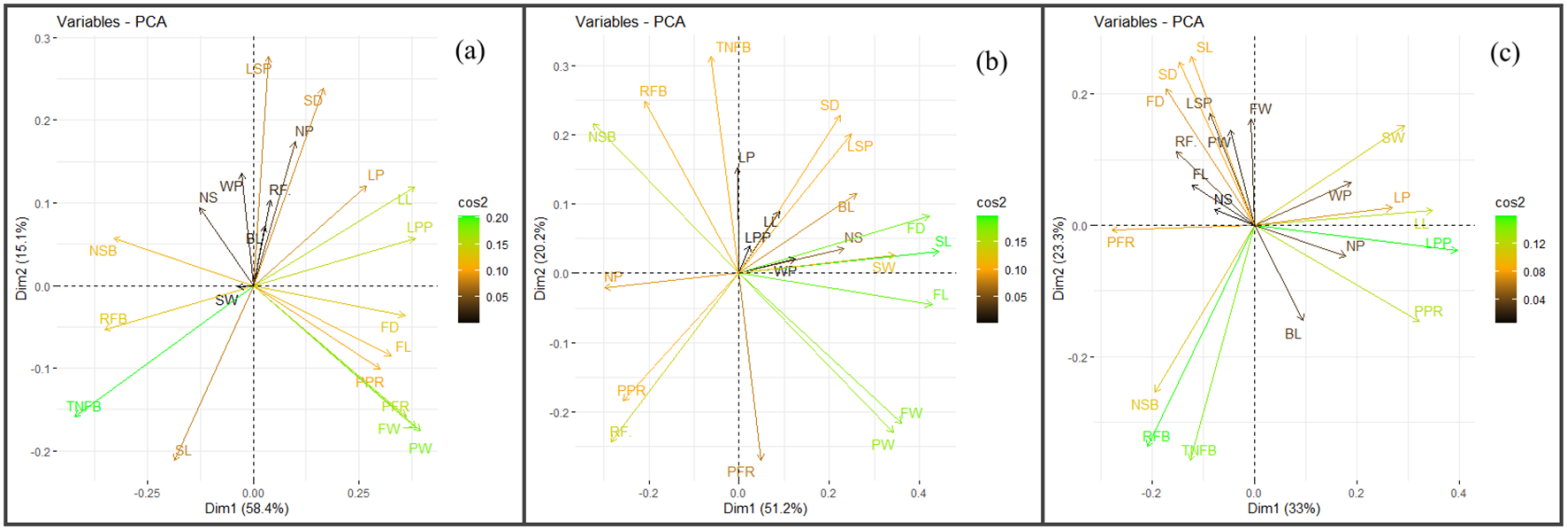
Principal component analysis (PCA) biplots of tissue culture (TC) and offshoot (OS) plants belonging to three date palm cultivars. **(a)** Kashuwari. **(b)** Gulistan. **(c)** Dedhi.

**Figure 2a** displays the results of the PCA on the morphological data of OS and TC plants of date palm cv. Kashuwari. The PCA revealed two PCs that explained 73.5% of the total variation in the data (**Supplementary Table S1**). The first two PCs (PC1 and PC2) explained 58.4% and 15.1% of the total variation, respectively. The third and fourth PCs captured less variation (10.3% and 7.6%, respectively) and therefore were not further discussed. The loadings on Dim1 (PC1) showed that the majority of the variables (14) had a positive association with this component. The variables with high positive loadings on Dim1 were LL, LPP, PPR, LP, FL, FD, FW, PW, and PFR. The loadings on Dim2 showed that some of the variables (11) had a positive association with this component, while others (10) had a negative association. The variables with the highest positive loadings on Dim2 were LSP, NP, and WP.

**Figure 2b** shows the results of the PCA on the morphological data of OS and TC plants of date palm cv. Gulistan. The PCA revealed two PCs that explained 71.4% of the total variation in the data (**Supplementary Table S1**). The first two PCs (PC1 and PC2) explained 51.2% and 20.2% of the total variation, respectively. The third and fourth PCs revealed less variation (16.7% and 6.2%, respectively). The loadings on Dim1 (PC1) showed that the majority of the variables (14) had a positive association with this component. The variables with the highest positive loadings on Dim1 were SL, FL, FD, FW, and PW. The loadings on Dim2 showed that 14 of the variables had a positive association with this component, while the remaining variables had a negative association. The variables with the highest positive loadings on Dim2 were TNFB, RFB (Retained Fruit per Bunch), NSB, SD, and LSP.

**Figure 2c** shows the results of the PCA on the morphological data of OS and TC plants of date palm cv. Dedhi. The PCA revealed three PCs that explained 76.4% of the total variation in the data (**Supplementary Table S1**). The first three PCs (PC1, PC2, and PC3) explained 32.9%, 23.3%, and 20.1% of the total variation, respectively. The fourth and fifth PCs revealed less variation (10.6% and 7.7%, respectively). The loadings on Dim1 (PC1) showed that eight variables had a positive association with this component. The variables with the highest positive loadings on Dim1 were LL, LPP, PPR, LP, and SW. The loadings on Dim2 (PC2) showed that 14 of the variables had a positive association with this component, while the remaining variables had a negative association. The variables with the highest positive loadings on Dim2 were SL, SD, and FD.

## 4 Discussion

The TC technique has been used for the micropropagation of several plant species for experimental and commercial purposes (Beauchesne and Rhiss, 1979; Damasco et al., 1997; Kılınç et al., 2015). This technique was originally developed as an artificial asexual method to propagate plants across all *in vivo* reproductive barriers, with the expectation of generating phenotypically and genotypically identical progeny. However, the basic expectation of generating identical progeny failed to some extent when SVs or changes in fruit quality attributes were observed in plants (Sala et al., 1999). The detection of SVs or the field evaluation of TC-derived plants using phenotypic markers has remained a beneficial, easy-to-use, and cost-effective method (Mirani et al., 2019, 2022).

**Figure 3:**
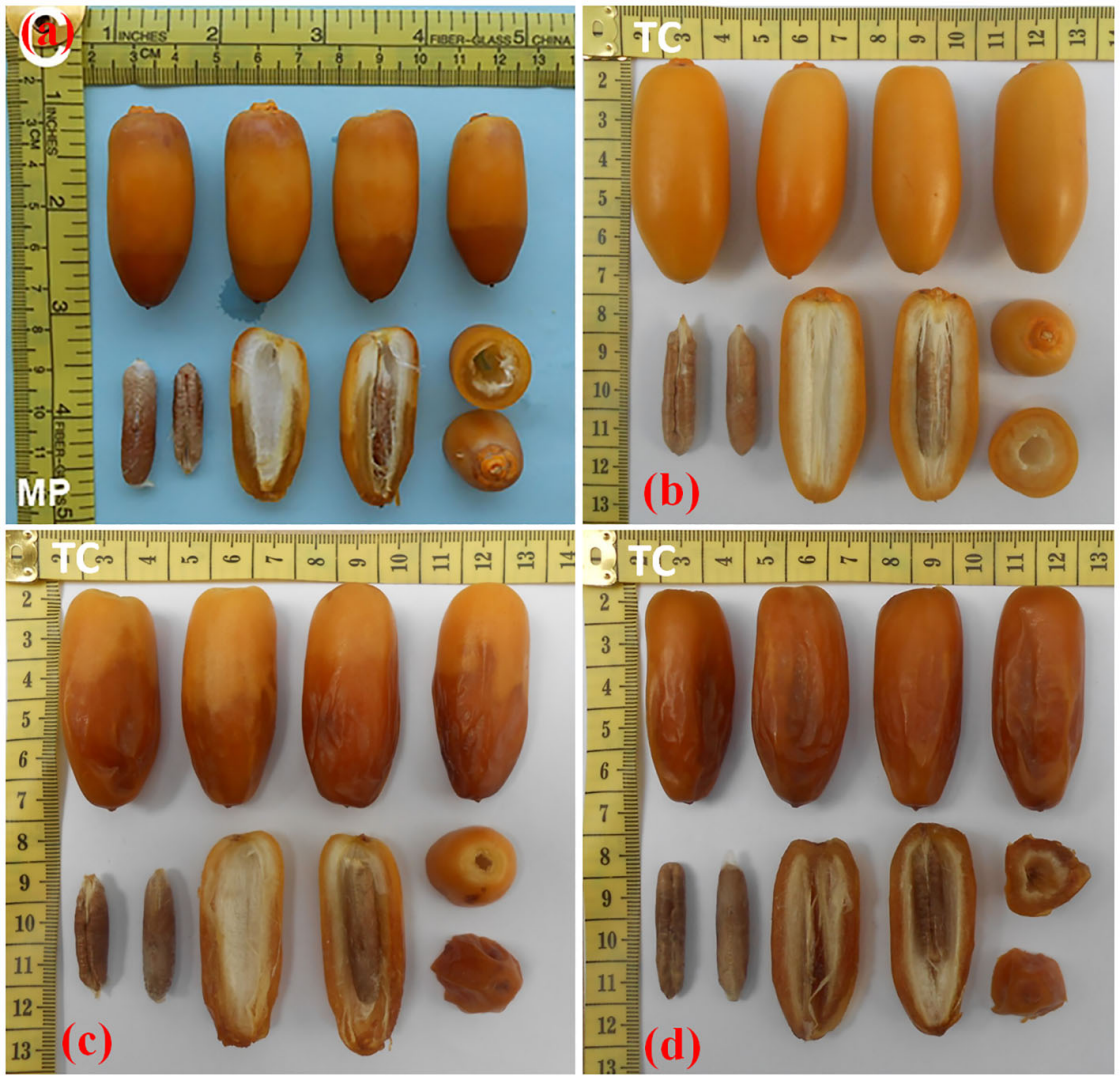
**(a)** Rutab-stage fruits from offshoot (OS)-grown mother plant of cv. Kashuwari. **(b)** Khalal-stage fruits from tissue cultivar (TC) plants of cv. Kashuwari. **(c)** Rutab-stage fruits from TC plants of cv. Kashuwari. **(d)** Tamar stage fruits from TC plants of cv. Kashuwari.

**Figure 4:**
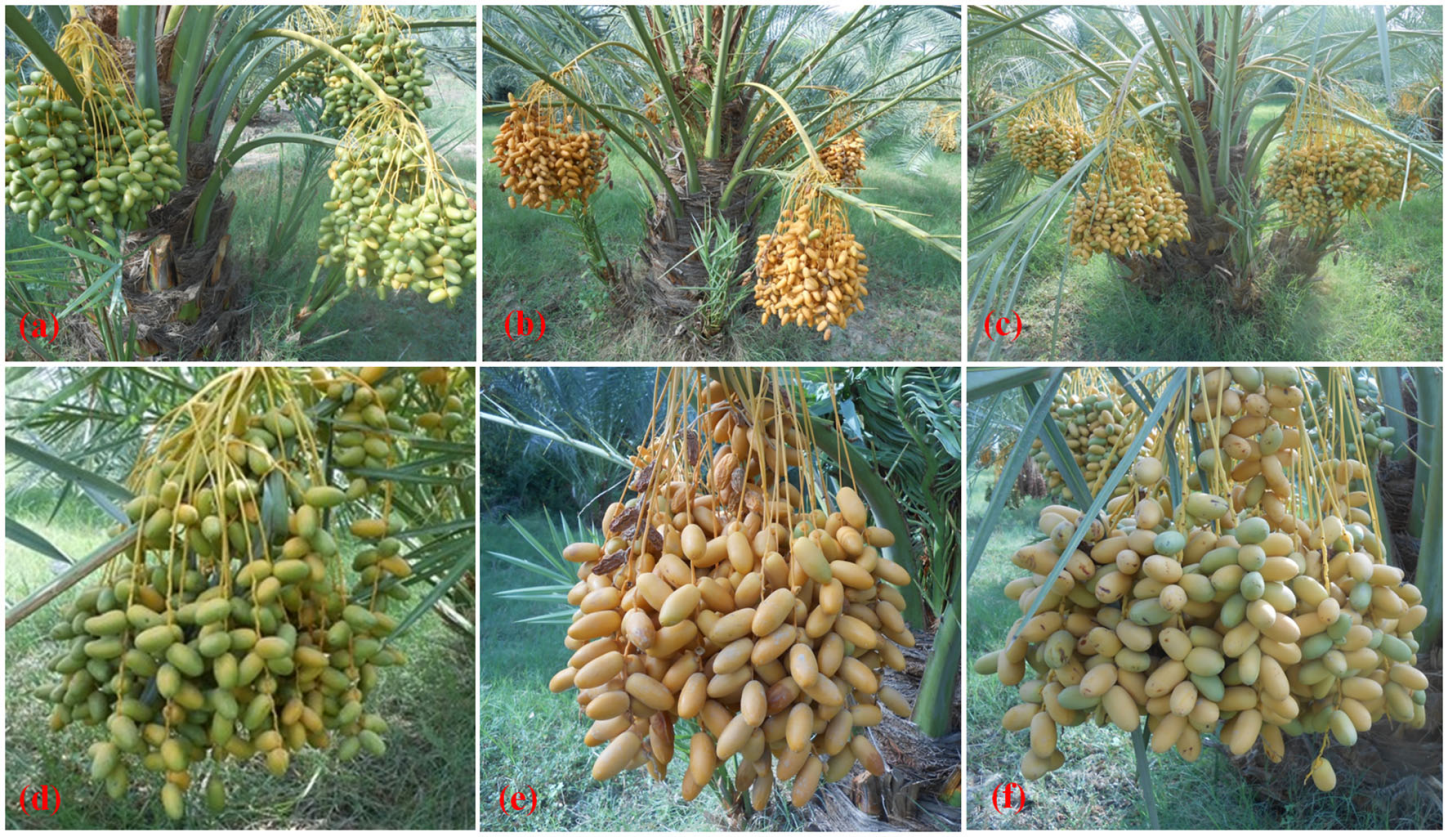
**(a–c)** Tissue-cultured date palms with fruits of cultivars Dedhi, Kashuwari, and Gulistan (*left* to *right*). **(d–f)** Fruit bunches of cultivars Dedhi, Kashuwari, and Gulistan (*left* to *right*).

**Figure 5:**
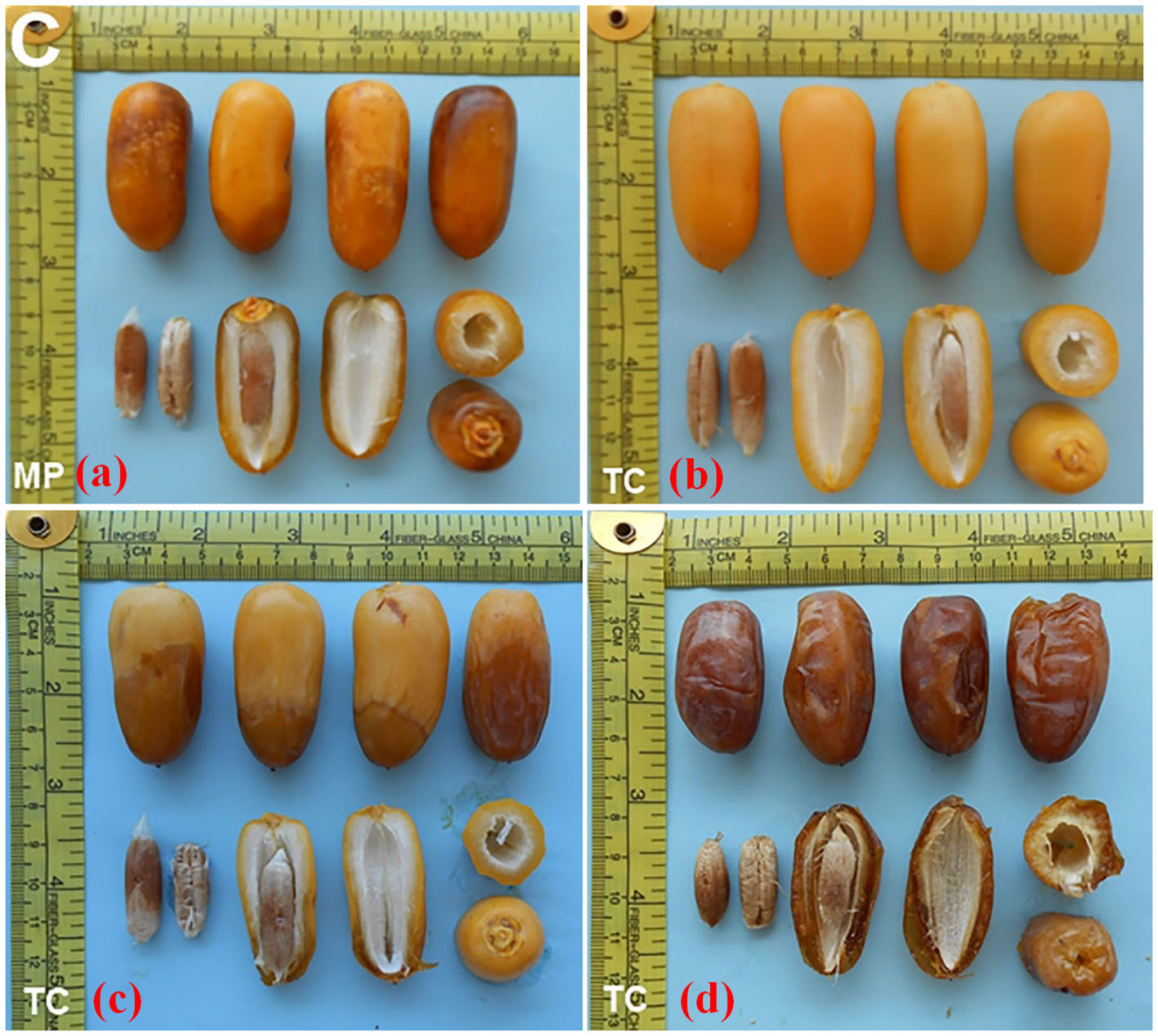
**(a)** Rutab-stage fruits of offshoot (OS)-grown mother plant of cv. Gulistan. **(b)** Khalal-stage fruits from tissue cultivar (TC) plants of cv. Gulistan. **(c)** Rutab-stage fruits from TC plants of cv. Gulistan. **(d)** Tamar stage fruits from TC plants of cv. Gulistan.

**Figure 6:**
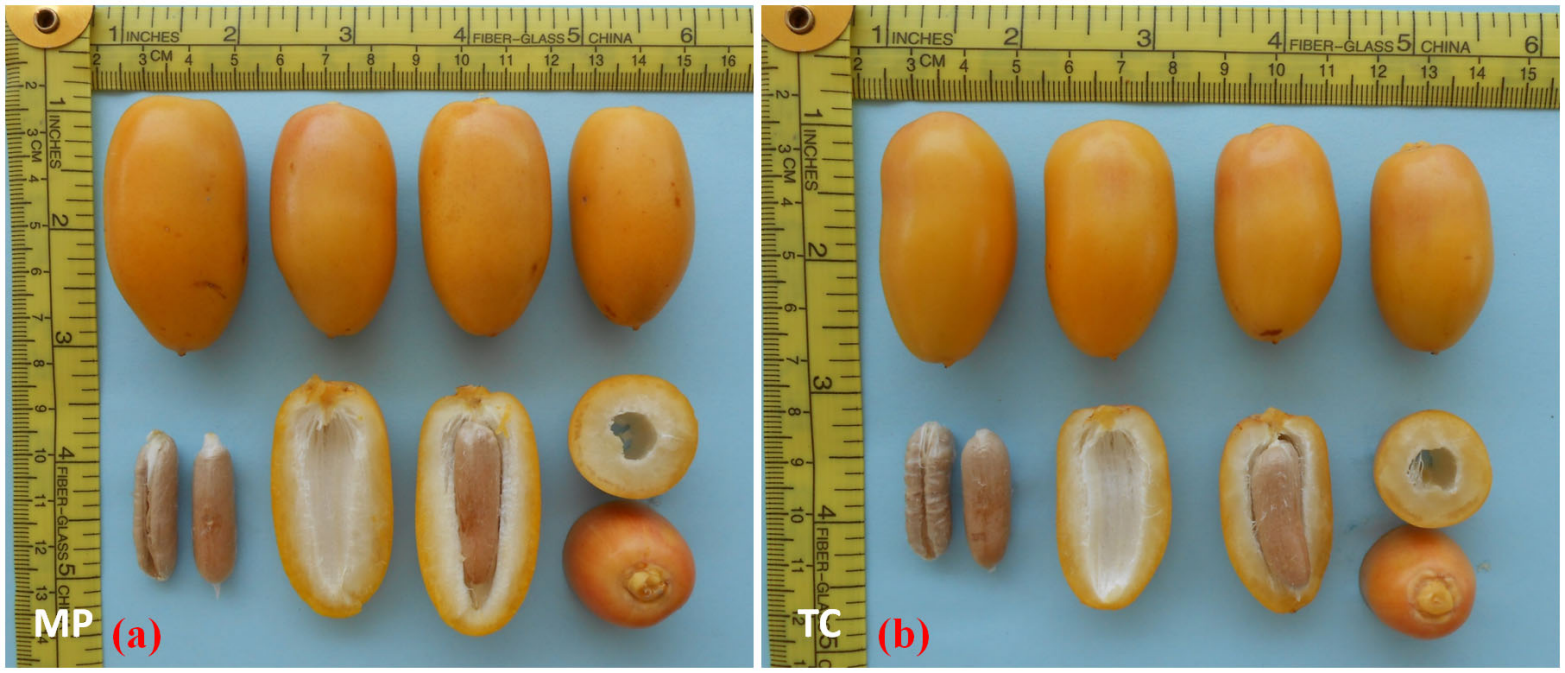
**(a)** Khalal-stage fruits from offshoot (OS)-grown mother plant of cv. Dedhi. **(b)** Khalal-stage fruits from tissue culture (TC) plants of cv. Dedhi.

In the current study, TC-derived plants of date palm cultivars Kashuwari, Gulistan, and Dedhi were compared with OS-grown mother plants to evaluate their vegetative, bunch, and fruit physical characteristics in the open field. Kashuwari is a semi-dry and early-season date palm cultivar from Khairpur, Sindh, Pakistan, with yellow fruits at the Khalal stage, consumed at the Rutab and Tamar stages, while the fruits are not edible at the Khalal stage due to the high quantity of tannins (**Figures 3a–d** and **4a, d**). Gulistan is a semi-dry date palm cultivar from Dera Ismail Khan, Khyber Pakhtunkhwa, Pakistan, with yellow fruits at the Khalal stage, consumed generally at the Rutab and Tamar stages. However, the fruit is also consumed at the Khalal stage due to its low tannin content and sweet taste (**Figures 4b, e**, **5a–d**). Dedhi is a soft type of date cultivar with yellow fruits having a light pinkish color just below the perianth, consumed at the Khalal stage. Therefore, fruit evaluation was carried out at the Khalal stage only (**Figures 4c, f**, **6a, b**).

The analysis of vegetative characteristics in the current study showed that there was no significant variation between TC and OS plants of cultivars Kashuwari, Gulistan, and Dedhi in the majority of the parameters. The differences in NP, LP, and WP in cv. Kashuwari, the LL in cv. Gulistan, and the LL, LPP, and LP in cv. Dedhi suggest that, in certain cases, the age of the plants and genetic or environmental factors can contribute to variability (Mirani et al., 2024). It is important to note that there was a considerable difference in age between TC and OS plants. The TC plants were 5 years old, whereas the OS plants were 20–40 years old. The juvenility of the TC plants likely played a role in the differences observed in traits such as vegetative growth and leaf size, as younger plants often prioritize vegetative development over reproductive traits. In contrast, the phenotypic stability of mature OS plants reflects a more stable expression of these characteristics.

The bunch characteristics were relatively stable across varieties, with no significant differences observed in BL, NSB, TNFB, and RF% in Kashuwari and Gulistan. However, Dedhi displayed significant differences in BL and RF%, indicating potential variation in the bunch-related traits. These differences could be attributed to environmental influences, such as microclimatic conditions, soil heterogeneity, or disease-related factors affecting fruit drop. Similar observations have been reported in previous studies, where environmental variables significantly impacted the bunch characteristics (El-Salhy et al., 2004). The fruit characteristics exhibited no significant differences between TC and OS plants in cv. Kashuwari, indicating consistency in the fruit dimensions and composition. In Gulistan, while the majority of parameters showed no significant variation, differences in FL, FD, and SL were observed. Dedhi, on the other hand, displayed uniformity in both fruit and seed characteristics. These results support the reliability of TC propagation for cv. Kashuwari and cv. Dedhi. However, the variation in FL, FD, and SL in cv. Kashuwari suggests genetic distinctions, pollination failure, or environmental influences that are affecting these traits. The contribution of SVs in cv. Gulistan further highlights the importance of monitoring genetic fidelity during the TC process, particularly in cultivars with known variability (Mirani et al., 2020, 2024).

Similar results have been observed in previous studies. For example, Smith and Aynsley (1995), in their study on the field evaluation of TC-derived date palm plants of cv. Barhi reported that the plants started bearing fruit within 4 years of planting in the field, with the fruits being similar in size to those of Barhi but were indistinguishable from the fruits of their OS-derived plants. Mirani et al. (2022) performed a field evaluation and genetic stability analysis of TC-derived date palm cv. Dedhi and observed true-to-type fruiting in the date palms; however, some phenotypic deformities were noted in the inflorescences during the initial fruiting up to 1–2 years, which reverted to normal phenotypes. Mirani et al. (2019) reported on the reversion of the phenotypic abnormalities to normal phenotypes in TC-derived date palm cultivars Kashuwari and Gulistan. The results of several other reports (Cohen et al., 2003, 2004) are in agreement with those reported by Mirani et al. (2019). The results of the current study are also in agreement with previously reported results on phenotypic abnormalities that are reversible to normal phenotypes and are not permanent genetic variations. However, this study focused on the vegetative, bunch, and fruit physical characterization of TC-derived cultivars of date palm, which confirmed the normal phenotype after 2 years of field planting.

Several other studies (Omar et al., 2014; Merwad et al., 2015; Abd-Elhaleem et al., 2020; Zargari et al., 2023) on the effect of pollen type on fruit properties of TC-derived and OS-grown date palm cultivars have shown its significant effect on fruit quality. However, in the field evaluation of the TC-derived date palm cultivars (Kashuwari, Gulistan, and Dedhi), the temporary phenotypic abnormalities reverting to normal phenotypes indicate that these are not the effects of the pollen type used for pollination. Mohammadi et al. (2017) also reported fruit abnormalities in the TC-derived date palm cv. Barhi caused by pollen type. Solangi et al. (2023a) conducted a detailed study on the micropropagation and field evaluation of TC-derived date palm cultivars Samany and Bertamoda, which confirmed normal vegetative, flowering, and fruiting characteristics of the plants. Solangi et al. (2023b), in another study conducted on the micropropagation and field transfer of TC-derived date palm cv. Barhi observed normal vegetative, flowering, and fruiting characteristics.

In general, a change in environmental conditions affects the expression of the phenotypic characteristics used in the evaluation of a plant species. Notable variations have been observed in the vegetative growth, bunch characteristics, fruit setting, and physical characteristics of fruits of different date palm cultivars grown under different climatic conditions, implying that the phenotypic characteristics of a date palm cultivar may differ from region to region and under various ecological and physiological conditions, such as the method of pollination, fertilization procedures, watering schemes, and soil conditions, along with the age factor (Azeqour et al., 2002; El-Salhy et al., 2004; Vij et al., 2005; Alkhateeb, 2008; Hasnaoui et al., 2011; Faissal et al., 2013). As an example, the intra-cultivar stability of six phenotypic characteristics in date palm cultivars (Deglet Noor, Alig, and Kintichi) reported under oasis stress from Tunisia (Hammadi et al., 2009) showed a high degree of independence between the phenotypic data and the geographical origin of the cultivars.

The results were further analyzed using the *k*-means clustering method. The *k*-means clustering method produced three main clusters, one for each date palm cultivar. Except for one TC plant of cv. Gulistan, all of the TC plants and their OS plants were placed in their respective clusters, demonstrating the phenotypic true-to-typeness of the TC plants with their respective mother (OS) plant. However, the clustering of a TC plant of cv. Gulistan with cv. Dedhi suggests a possible SV or environmental influence. Such anomalies have been reported in previous studies (Mirani et al., 2020, 2019), particularly in cultivars prone to genetic variability during micropropagation. Excessive leaf growth, excessive vegetative growth, and deformed OS production, among other types, were the most prominent variations found in cv. Gulistan in previous studies (Mirani, 2019; Mirani et al., 2019, 2020).

PCA was also conducted on the three date palm cultivars to visualize the factors contributing to any variation. PCA is a multivariate analysis technique that provides a comprehensive representation of all evaluated variables (El-Sanatawy et al., 2021; Hamad et al., 2022; Gunsilius and Schennach, 2023; Mansour et al., 2023). The morphological data used for the PCA revealed that the first two PCs explained a significant proportion of the total variation in all three date palm cultivars, ranging from 71.4% to 76.4%. The variables with the highest positive loadings on the first two components represented the most important morphological characteristics contributing to the differences between TC and OS plants. These findings align with previous research indicating that the combination of the PCA and clustering methods is effective in reducing data dimensionality and uncovering patterns in multivariate datasets. For instance, Kadri et al. (2021) employed PCA to explore the association between the phenotypic attributes in different Tunisian date palm cultivars, highlighting significant trait contributions to the total divergence. Although there were very few variables that contributed to significant variation between TC and OS plants in the current study, their impact cannot be ignored. Haider et al. (2015) observed phenotypic variation among Pakistani date palm cultivars collected from different sources, further supporting the role of phenotypic assessment in cultivar differentiation. In cv. Kashuwari, the leaf- and fruit-related variables contributed the majority of the variation between TC and OS plants in cv. Gulistan, the bunch- and fruit-related traits were the primary sources of variation. Similarly, in cv. Dedhi, the leaf- and fruit-related variables contributed the the majority of the observed differences. Comparable findings were reported by Solangi et al. (2023a, b) during the field performance evaluation of inflorescence explant-derived date palm cultivars Samany, Bertamoda, and Barhi. PCA can not only objectively select the resources with excellent comprehensive quality but also make an appropriate evaluation of the various resources according to the scores. This approach prevents the inadvertent elimination of valuable genetic material while facilitating a systematic assessment and ranking of the different resources (Zhang et al., 2024).

The results of the current study and those published previously (Abul-Soad and Jatoi, 2014; Jatoi et al., 2015; Mirani et al., 2019; Solangi et al., 2023a, b) suggest that the majority of the variations between TC and OS plants were statistically non-significant. However, minor variations due to genetic, environmental, and age-related factors underscore the need for ongoing evaluation to ensure long-term stability. This study highlights the relationship between these factors and provides a basis for refining the TC protocols for broader application in date palm propagation and cultivation.

## 5 Conclusion

Field evaluation of micropropagated date palm cultivars was successfully carried out to evaluate and compare the vegetative, bunch, and fruit characteristics of TC-derived date palms with OS-grown mother plants. The fruits produced in each TC-derived cultivar were similar to those obtained from OS plants in terms of size, shape, color, and taste. Very few significant variations were observed between TC and OS plants with regard to morphological characteristics, which might have been due to the significant difference in age between these TC and OS plants. The PCA revealed that the majority of the variability was explained by key traits, with the leaf and fruit characteristics contributing the most. The *k*-means clustering test confirmed the phenotypic true-to-typeness of the TC plants, except for one TC plant of cv. Gulistan, which was likely affected by SV or environmental factors. This suggests that the current micropropagation protocol using juvenile inflorescence explants is suitable for the production of morphologically similar plants. The current protocol, optimized up to fruiting of the micropropagated plants, can be applied for micropropagation and conservation of other cultivars of date palms with a limited number of OS growing in Pakistan and around the world.

## Data Availability

The original contributions presented in the study are included in the article/**Supplementary Material**. Further inquiries can be directed to the corresponding authors.
