# Supplementary material for: Field evaluation of tissue culture-derived and offshoot-grown date palm cultivars: a comparative analysis of vegetative and fruit attributes
Source: Front Plant Sci. 2025 Apr 24;16:1516983. doi: 10.3389/fpls.2025.1516983 (PMC12058846; doi:10.3389/fpls.2025.1516983)
Supplement: Supplementary file 1 [file Table1.docx]

**Supplementary table 1.** Loadings of first three PCs for Vegetative, Bunch and fruit physical variables of date palm cvs. Kashuwari, Gulistan and Dedhi

|  | **Kashuwari** | | | **Gulistan** | | | **Dedhi** | | |
| --- | --- | --- | --- | --- | --- | --- | --- | --- | --- |
| **Variables** | **PC1** | **PC2** | **PC3** | **PC1** | **PC2** | **PC3** | **PC1** | **PC2** | **PC3** |
| **LL** | 0.3 | 0.184 | 0.118 | 0.071 | 0.113 | 0.405 | 0.364 | 0.027 | 0.107 |
| **LSP** | 0.028 | 0.428 | -0.356 | 0.198 | 0.255 | 0.141 | -0.092 | 0.211 | 0.324 |
| **LPP** | 0.3 | 0.089 | 0.204 | 0.02 | 0.051 | 0.435 | 0.415 | -0.048 | -0.006 |
| **PPR** | 0.236 | -0.157 | 0.361 | -0.206 | -0.233 | 0.187 | 0.337 | -0.183 | -0.242 |
| **NS** | -0.099 | 0.145 | 0.263 | 0.186 | 0.046 | -0.245 | -0.082 | 0.03 | -0.192 |
| **NP** | 0.077 | 0.269 | 0.279 | -0.238 | -0.026 | -0.261 | 0.187 | -0.059 | -0.326 |
| **LP** | 0.209 | 0.186 | 0.337 | -0.003 | 0.194 | -0.175 | 0.282 | 0.033 | 0.23 |
| **WP** | -0.021 | 0.209 | 0.278 | 0.1 | 0.026 | 0.237 | 0.197 | 0.081 | 0.03 |
| **BL** | 0.021 | 0.111 | 0.041 | 0.208 | 0.145 | -0.11 | 0.1 | -0.181 | -0.272 |
| **NSB** | -0.258 | 0.089 | 0.245 | -0.258 | 0.273 | 0.218 | -0.203 | -0.316 | -0.009 |
| **RFB** | -0.276 | -0.082 | 0.098 | -0.167 | 0.314 | 0.224 | -0.219 | -0.42 | 0.093 |
| **TNFB** | -0.331 | -0.245 | 0.159 | -0.05 | 0.397 | 0.223 | -0.132 | -0.446 | 0.085 |
| **RF.** | 0.032 | 0.159 | -0.05 | -0.226 | -0.308 | -0.041 | -0.16 | 0.139 | -0.004 |
| **FL** | 0.255 | -0.131 | -0.284 | 0.341 | -0.057 | 0.134 | -0.128 | 0.077 | -0.247 |
| **FD** | 0.281 | -0.056 | -0.006 | 0.336 | 0.105 | 0.048 | -0.181 | 0.258 | 0.146 |
| **FW** | 0.301 | -0.266 | -0.083 | 0.288 | -0.274 | 0.122 | -0.007 | 0.201 | -0.411 |
| **PW** | 0.309 | -0.271 | -0.054 | 0.273 | -0.291 | 0.146 | -0.05 | 0.18 | -0.424 |
| **PFR** | 0.283 | -0.244 | 0.172 | 0.039 | -0.343 | 0.301 | -0.292 | -0.008 | -0.307 |
| **SL** | -0.146 | -0.327 | 0.177 | 0.353 | 0.04 | -0.043 | -0.13 | 0.32 | -0.03 |
| **SD** | 0.13 | 0.368 | -0.033 | 0.179 | 0.289 | -0.187 | -0.154 | 0.309 | 0.109 |
| **SW** | -0.029 | -0.003 | -0.314 | 0.275 | 0.034 | -0.178 | 0.306 | 0.189 | -0.001 |
| **Eigenvalue** | 1.63 | 4.20 | 2.88 | 1.58 | 6.22 | 5.16 | 9.16 | 6.46 | 5.59 |
| **Percentage accounted** | 58.4 | 15.1 | 10.3 | 51.2 | 20.2 | 16.8 | 33 | 23.3 | 20.1 |
